# Tissue Healing in Hemicraniectomy

**DOI:** 10.7759/cureus.29260

**Published:** 2022-09-17

**Authors:** Ntenis Nerntengian, Tammam Abboud, Adam Stepniewski, Gunther Felmerer, Veit Rohde, Levent Tanrikulu

**Affiliations:** 1 Neurological Surgery, University Medical Center of Göttingen, Göttingen, DEU; 2 Trauma Surgery, Orthopedics and Plastic Surgery, University Medical Center of Göttingen, Göttingen, DEU

**Keywords:** emergency neurosurgery, retromastoidal (rmf) incision, dandyflap, tissue healing, hemicraniectomy

## Abstract

Introduction

Decompressive hemicraniectomy (DHC) is a last-resort treatment for refractory intracranial hypertension. Perioperative morbidity is associated with high risks of wound healing disturbances (WHD). Recently, a retromastoidal frontoparietooccipital (RMF) incision type was performed to avoid healing disturbance due to enhanced tissue flap perfusion compared to the classical reverse “question mark” (“Dandy flap”) incision. The goal of this study was to analyze the details of tissue healing problems in DHC.

Materials and methods

A total of 60 patients who underwent DHC were retrospectively analyzed. In 30 patients the “Dandy flap” incision (group A) and in 30 patients the RMF incision (group B) was made. Since no evidence-based data for the incision type that favors better wound healing exists, the form of incision was left at the surgeon´s discretion. Documentation of the patients was screened for the incidence of WHD: wound necrosis, dehiscence, and cerebrospinal fluid (CSF) leakage. Patient age, the time interval from surgery until the appearance of WHD, the length of surgeries in minutes, and the indications of the DHC were analyzed. A Chi-square test of independence was performed to examine the relationship between the incision type and the appearance of WHD with the statistical significance level set at p<0.05. The mean age of the patients, the mean time interval from surgery until the occurrence of WHD, and the mean length of the surgery between the two groups were compared using an independent sample t-test with the statistical significance level set at p<0.05.

Results

The most common indication for DHC in both groups was malignant MCA infarction (n=20, 66.6% for group A and n=16, 53.3% for group B). CSF leakage was 20% of the most frequent WHD in each group. Wound necrosis was observed only in group A. Although group B showed 13.3% fewer WHD than group A, this difference was not statistically significant. There was no statistically significant difference in the time range between surgery and the occurrence of WHD between the two groups. The length of surgery in group B was significantly shorter than in group A (120.2 mins vs. 103.7 mins).

Conclusion

A noticeable trend for reduced WHD was observed in the patient group using the RMF incision type although the difference was not statistically significant. We praise that the RMF incision allows an optimized skin-flap vascularization and, thereby, facilitates better wound healing. We were able to show a statistically shorter length of surgery with the RMF incision in contrast to the classic “Dandy flap” incision. Larger multicenter studies should be implemented to analyze and address the major advantages and pitfalls of the routinely applied incision techniques.

## Introduction

Decompressive hemicraniectomy (DHC) is a life-saving procedure in patients with increased intracranial pressure (ICP) of vascular etiology (usually malignant middle cerebral artery infarction) or traumatic brain injury [1,2]. However, the morbidity of DHC is remarkably high and tissue healing complications amount to up to 40% for a major part of this morbidity, prolonging the duration of hospitalization and delaying cranioplasty [3,4]. Various incision techniques are used for DHC: the classic reverse “question mark” incision, also known as “Dandy flap” or “trauma flap”, and the alternative posterior “question-mark” incision, which is defined as the retromastoidal frontoparietooccipital (RMF) incision type [5,6]. The impact of different incision techniques on tissue healing in the immediate postoperative period after DHC is sparsely analyzed in detail and fewer studies deal with healing issues after cranioplasty. The core objective of our investigation was to determine if there was any difference in the incidence of tissue healing problems after DHC was performed with the aforementioned different incision characteristics.

## Materials and methods

**Figure 1:**
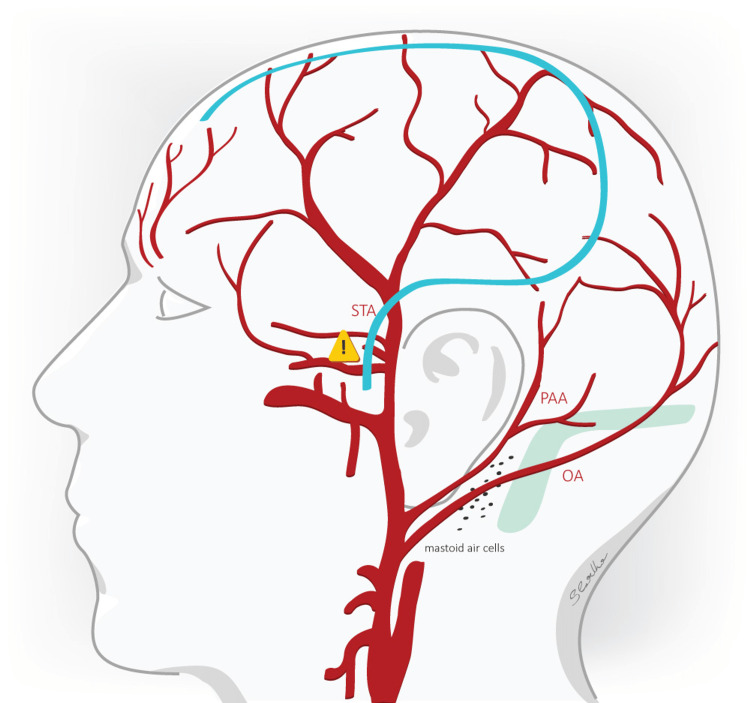
The reverse “question mark” or “Dandy flap” incision The reverse “question mark” (“Dandy flap”) incision is superimposed on the vasculature of the lateral scalp. The STA provides the main arterial blood supply of the skin flap created and is prone to injury in the emergency operating setting of a hemicraniectomy. References [7,8] STA: superficial temporal artery, PAA: posterior auricular artery, OA: occipital artery, blue line: incision.

**Figure 2:**
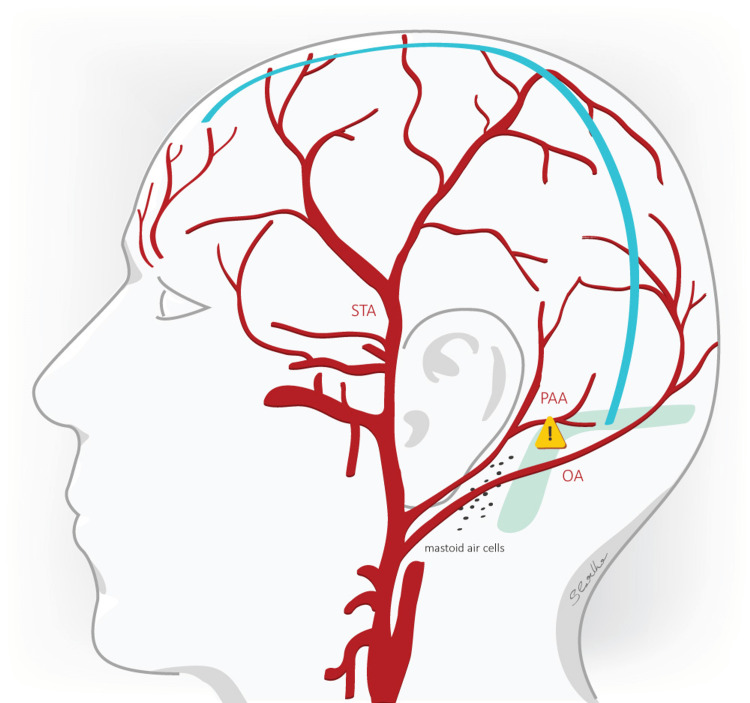
RMF incision With the retromastoidal frontoparietooccipital (RMF) incision, not only the STA but the PAA is also providing arterial supply to the skin flap created. The STA being the main arterial supply of the skin flap is protected with this type of incision. Care must also be taken to preserve the OA. References [7,9,10] STA: superficial temporal artery, PAA: posterior auricular artery, OA: occipital artery, blue line: incision.

Ethics approval for our retrospective study was granted by the Ethics Commission of the University Medical Center of Göttingen. We retrospectively analyzed 131 patients who underwent DHC between January 2018 and November 2020 in our institution. Patients with either “Dandy flap” or RMF incision who survived at least 14 days postoperatively were included in our analysis. DHC following osteoplastic craniotomies were excluded as they required different incision types based on the prior surgery. Due to lacking evidence-based data on which incision type favors better wound healing, the type of incision was left at the surgeon´s discretion. The dura was left open in all patients. A total of 60 patients who met the above criteria were included. In 30 patients “Dandy flap” incision (Figure 1) (group A) and in 30 patients RMF incision (Figure 2) (group B) was applied.

The medical documentation of the patients was screened for the occurrence of WHD such as wound necrosis, wound dehiscence, and CSF leakage. The age of the patients, the time interval from surgery until the appearance of tissue healing problems, the length of the operations in minutes, and the indications of the DHC were also analyzed. A Chi-square test of independence was performed to examine the relationship between the incision type and the appearance of WHD with the statistical significance level set at p<0.05. The mean age of the patients in years, the mean time interval from surgery to the occurrence of wound healing disturbances in days, and the mean length of surgery in minutes between the two groups were compared using an independent sample t-test between the mean values with the statistical significance level also set at p<0.05. 

## Results

The main indication in both groups, 60% in total (n=36), was malignant middle cerebral artery infarction (Table 1). Other indications were traumatic brain injury (n=11, 18.3%), spontaneous intracerebral hemorrhage (n=7, 11.7%), aneurysmatic subarachnoid hemorrhage (n=4, 6.7%) and venous sinus thrombosis (n=2, 3.3%). The mean patient age was 56.5 years (57.2 years in group A and 55.8 years in group B). There was no statistically significant difference between the mean age of the two groups (p=0.721). 

**Table 1:** Indication for decompressive hemicraniectomy procedure MCA: middle cerebral artery

Indication	Group A (n=30)	Group B (n=30)	Total (%)
Malignant MCA infarction	20	16	36 (60)
Traumatic brain injury	4	7	11 (18.33)
Spontaneous intracerebral hemorrhage	3	4	7 (11.67)
Aneurysmal subarachnoidal hemorrhage	2	2	4 (6.67)
Cerebral venous sinus thrombosis	1	1	2 (3.33)

A total of 20 patients developed a WHD (33.3%): 12 out of 30 patients (40%) in group A and 8 out of 30 patients (26.7%) in group B. Although group B showed 13.3% fewer WHD than group A, there was no statistically significant relation between the incision type and the appearance of WHD (X2 (1, N=60)=1.2, p=0.273) (Table 2).  

**Table 2:** Examined parameters of the groups

	Group A (n=30)	Group B (n=30)	p-value
Mean age in years	57.16	55.76	0.721
Wound healing disturbance (%)	12 (40%)	8 (26.67%)	0.278
Surgery duration in mins	120.2	103.73	0.029
Mean days from surgery to the occurrence of wound healing disturbance	27.3	18.87	0.165

The most frequent WHD in both groups was CSF leakage with a total occurrence of 12 (60%) cases (Table 3). These cases were primarily treated with the placement of a lumbal drain with or without suturing the skin at the fistula point in a sterile fashion. Persisting CSF leakage was treated with the revision of the wound. Other WHD included wound dehiscence (n=4, 20%) and wound necrosis (n=4, 20%). Wound necrosis occurred only in group A. The mean duration of surgery in group A was 120.2 min and in Group B was 103.7 min. The mean time interval between surgery and occurrence of WHD was 23.9 days: 27.3 days in group A and 18.9 days in group B (p=0.165). The length of DHC performed with the RMF incision was significantly shorter than the DHC performed with the Dandy flap incision (p=0.029). Except for one case of exposed mastoid air cells in the group of DHC using the RMF incision, we did not observe any other intraoperative complications.

**Table 3:** Type of wound healing disturbances in each group CSF: cerebrospinal fluid

Complications	Group A (n=30)	Group B (n=30)	Total
CSF leakage	6	6	12 (60%)
Dehiscence	2	2	4 (20%)
Necrosis	4	0	4 (20%)

## Discussion

DHC is a well-established procedure to treat increased ICP which is refractory to conservative management [11]. The rate of postoperative complications such as seizures, infection, subdural hygromas and hydrocephalus is high [12]. Grindlinger et al. report four cases with postoperative CSF leak and three cases with meningitis in a series of 31 DHCs performed in the setting of severe traumatic brain injury, which correspond to a rate of approx. 22.6% [13]. Sughrue et al. report postoperative wound complications including wound infection, CSF leak, and wound dehiscence in 35% of their cohort of 98 patients [4].

In our analysis, we saw a noticeable trend of decreased tissue healing disturbances with the RMF incision as opposed to the "Dandy flap" incision; possibly, the limited number of patients prevented that a statistically significant result was observed. Apart from the meticulous surgical technique at the stage of wound closure and postoperative wound management, recent literature emphasizes that sufficient skin flap vascularization plays a key role in supporting tissue healing [7,9,10]. Both, the RMF and "Dandy flap" incisions, create a combination of different tissue layers that cover the lateral aspect of the scalp where the main arterial supply is provided by the superficial temporal artery (STA) with its frontal and parietal branches.

The inadvertent injury of the STA in an emergency setting (vessel diameter at the bifurcation point to frontal and parietal branch is approx. 1.8-2.7 mm [14]) will negatively influence the blood supply of the musculocutaneous tissue flap making it dependent mainly on the smaller supraorbital und supratrochlear arteries (vessel diameter 0.84-0.87 mm and 0.8 mm respectively [15]), from which the supraorbital artery anastomoses with the frontal branch of the STA [14]. In contrast to the "Dandy flap" incision, the RMF incision additionally includes the patency of the posterior auricular artery and its branches for the perfusion of the created flap. We consider that the intact STA along with the supply from the patent posterior auricular artery is the main reason for the reduced rate of tissue healing problems compared to the "Dandy flap" incision.

Preserving the occipital artery in the retromastoidal region using the blunt dissection technique [9] is important as this will maintain the blood supply to the skin edges directly posteromedial of the RMF incision. The role of vascularization of the flap is emphasized by the fact that CSF leakage and wound dehiscence occurred with equal frequency in both incision types, whereas wound necrosis was seen only in patients with "Dandy flap" incision types. Care must be taken at the dorso-basal portion of the craniectomy to avoid inadvertent injury to the transverse or the sigmoid sinus around their junction and mastoid air cells should be sealed with bone wax when they are unintentionally exposed [7]. As the RMF incision compared to the "Dandy flap" incision exposes the retroauricular calvarial area, we find the aforementioned risks of sinus injury and mastoid cell exposure higher in the RMF incision than in the "Dandy flap" incision.

Additionally, at this part of the surgical site, one should also exercise caution not to damage the external auditory canal during skin flap preparation [10]. In our series, we did not encounter any incident of injury to venous sinuses or to the external auditory canal. One case with mastoid air cell exposition was adequately managed with bone wax and no wound healing disturbance appeared in the postoperative follow-up of this patient. The musculocutaneous flap is larger in RMF incisions in comparison to the Dandy flap incision. We observed a statistically significant shorter length of surgery when the RMF incision was made despite the perception of more laborious temporal muscle preparation and osteoclastic bone removal in these cases.

The findings of Veldeman et al. in a large retrospective study including 186 patients showed statistically significant fewer surgical site infections after cranioplasty, following DHC done with RMF incision as opposed to DHC performed with the "Dandy flap" incision (6.3% vs. 14%) [6]. In accordance with our results, Dowlati et al. detected in a recent study a lower rate of wound complications with the RMF incision compared to DHC performed with the "Dandy flap" incision (8.3% vs. 14%) in a larger sample of 106 patients also without reaching statistically significant difference [8].

The main limitation of our study was the relatively limited number of the patient population. In addition, we acknowledge that the majority of surgeons are more familiar with the "Dandy flap" incision and that the adaptation to the RMF incision may require some time. Further larger prospective randomized multicenter studies are necessary to investigate the advantages and disadvantages of both incision techniques. 

## Conclusions

The results of our study show, albeit not statistically significant, a noticeable trend towards less tissue healing disturbances with the use of RMF incision in DHC. We were able to show a statistically significant shorter length of surgery when this incision type was used. Multicenter study randomization is needed to evaluate further the effect of the skin incision type on postoperative tissue healing problems to obtain reproducible results.
